# Spontaneous regression of metastatic cancer cells in the lymph node: a case report

**DOI:** 10.1186/1756-0500-7-293

**Published:** 2014-05-13

**Authors:** Nayeon Choi, Jae Keun Cho, Chung-Hwan Baek, Young-Hyeh Ko, Han-Sin Jeong

**Affiliations:** 1 Department of Otorhinolaryngology - Head and Neck Surgery, Samsung Medical Center, Sungkyunkwan University School of Medicine, 50 Irwon-dong, Gangnam-gu, Seoul 135-710, Korea; 2 Departments of Pathology, Samsung Medical Center, Sungkyunkwan University School of Medicine, Seoul, Korea

**Keywords:** Neoplasms, Lymph nodes, Lymphatic metastasis, Spontaneous neoplasm regression, Cellular immunity

## Abstract

**Background:**

Spontaneous regression of a malignant tumor is the phenomenon of disappearance of cancer cells without any treatments and it can be induced by an enhanced tumor-targeting immune response. However, there has not been a comprehensive immunological overview to compare the tumor-regressed lymph nodes and metastatic lymph nodes in the same patient.

**Case presentation:**

We conducted a histologic analysis of various immune cells in an Asian female patient with buccal cancer (squamous cell carcinomas), in which the spontaneous regression of metastatic lymphadenopathy was confirmed by surgical pathology. The immune cell profiles between the metastatic nodes and the tumor-regressed nodes were compared. Tumor regression was confirmed by hematoxylin & eosin and cytokeratin/Ki-67 staining. Distinct differences were observed in Foxp3(+) regulatory T (Treg) cells and CD56(+) natural killer (NK) cells; a higher density of Foxp3(+) Treg cells was found in metastatic lymph nodes and more infiltration of CD56(+) NK cells in tumor regressed lymph nodes. Other immune cell populations (CD4, CD8, CD20, CD68, CD86, CD123, CD11c, and mannose receptor) showed no discernible differences in marker expression in the nodes examined.

**Conclusion:**

Less recruitment of Treg and high infiltration of NK cells were key features in tumor-regressed lymph nodes. Modulation of Treg or NK cells may be a good therapeutic method to control lymph node metastasis.

## Background

Spontaneous regression of a malignant tumor is the phenomenon of disappearance of cancer cells without any treatment [1,2]. Even though this event seems to be rare, it has been observed for thousands of years [3]. Spontaneous regressions are known to be related to acute infections, vaccine therapy, surgical removal of the malignant tumor and the taking of herbal medicines [4]. And these phenomena are more common in pediatric embryonal tumors, and breast and skin cancers [4].

Of note, the immunological characteristics of these tumors have drawn attention to the main underlying mechanisms of spontaneous regression. Previous studies on spontaneous regression have involved investigations of various immunologic markers for natural killer cells, macrophages and dendritic cells, that could be involved in enhanced tumor targeting immune responses [5,6]. However, there has not been a comprehensive immunological overview comparing lymph node metastasis and spontaneously regressed metastasis in the lymph nodes of the same patient. We think it is very important because the immune cell profiles are quite variable between individual hosts, depending upon age [7], hormonal status [8-10], environmental cues [11], nutrition [12] and even bio-behavior [13].

Thus, we conducted an immunological study in one patient with buccal cancer (squamous cell carcinoma), in which spontaneous regression of metastatic lymphadenopathy was confirmed by surgical pathology. We expect that our study will contribute to the understanding of the underlying mechanisms of spontaneous regression of solid cancers, and hopefully, the findings will suggest new immunological methods of preventing or treating cancer or corresponding lymph node metastasis.

## Case presentation

### Clinical course

**Figure 1:**
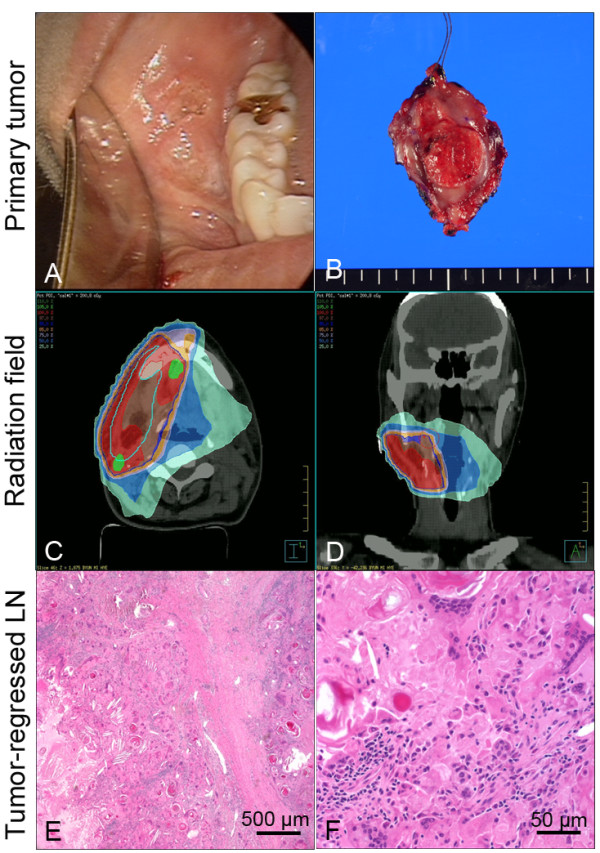
**Clinical findings and pathology of a tumor-regressed lymph node. (A)** Ulcero-fungating mass in the right buccal area, which proved to be a squamous cell carcinoma. **(B)** Gross photo of the excised primary tumor. **(C-D)** Radiation fields for adjuvant radiotherapy, which did not include the left lower neck. **(E-F)** Pathology view with hematoxylin and eosin staining of the tumor-regressed lymph node.

A 52-year-old Asian female patient was admitted to our clinic, with complaints of right cheek pain and an ulcerative protruding mass lesion in the right buccal area. We conducted punch biopsy for the buccal mass and this revealed well-differentiated squamous cell carcinoma. Computed tomography (CT), magnetic resonance imaging (MRI) and positron emission tomography/computed tomography (PET/CT) were conducted and these delineated a 2.9 × 2 × 1.4 cm buccal mass in the right lower cheek with right lymph node metastasis (level Ib, IIa and III), and there was no evidence of distant metastasis. We made a diagnosis of buccal cancer cT2N2bM0 (Figure 1A-B). She underwent wide surgical resection of the malignant tumor in the right buccal area, right comprehensive neck dissection (level I to V) and left selective neck dissection (level I to III). The defect that occurred after the tumor resection was reconstructed with an omental free flap. According to the pathologic TNM (tumor, node, metastasis) staging system, the final diagnosis was pT2N1 (right lymph node metastasis). Post-operative adjuvant radiotherapy was administered, covering the primary sites and regional nodes (right whole neck and left upper neck) (Figure 1C-D).

After the treatment mentioned above, we regularly checked the CT, MRI and PET/CT as scheduled in the outpatient clinic. During the follow-up period, intermittent swelling of the neck lymph nodes was observed. Therefore, we performed ultrasonography guided fine needle aspiration cytology to exclude recurrence, and there was no evidence of malignant tumor cells. She has remained in a disease-free state for 4 years. At post-treatment 4 years 6 months, a markedly enlarged lymph node in the contralateral lower neck (left level V) was found. CT and ultrasonography guided fine needle aspiration cytology showed regional lymph node recurrence, which was suggestive of metastatic squamous cell carcinoma, based on the necrotic features of the enlarged lymph nodes on the CT and the keratin debris on cytology (Additional file 1: Figure S1).

Salvage neck dissection was performed for these enlarged lymph nodes; however, surgical pathology revealed total regression of metastatic squamous cell carcinoma cells in the left level V lymph node and there was no evidence of malignancy in the other dissected lymph nodes (Figure 1E-F). Interestingly, the left level V lymph nodes were not involved in the previous surgery or radiation fields. In addition, the patient denied any self-remedy or other treatments.

### Immunohistochemistry findings

To further characterize this rare event, we performed immunohistochemistry on the various immune cells. The patient submitted written informed consent for each procedure and our Institutional Review Board approved the use of the archived tissues (Approval No. 2013-08-088-001). We compared the immune cell profiles among the metastatic node, non-tumor bearing node (from the first surgery) and the tumor-regressed nodes (from the second surgery). The antibodies for staining were cytokeratin, Ki-67, CD4, CD8, CD20, CD68, Mannose receptor, Foxp3, CD56, CD86, CD11c, and CD123 (Additional file 2: Table S1).

**Figure 2:**
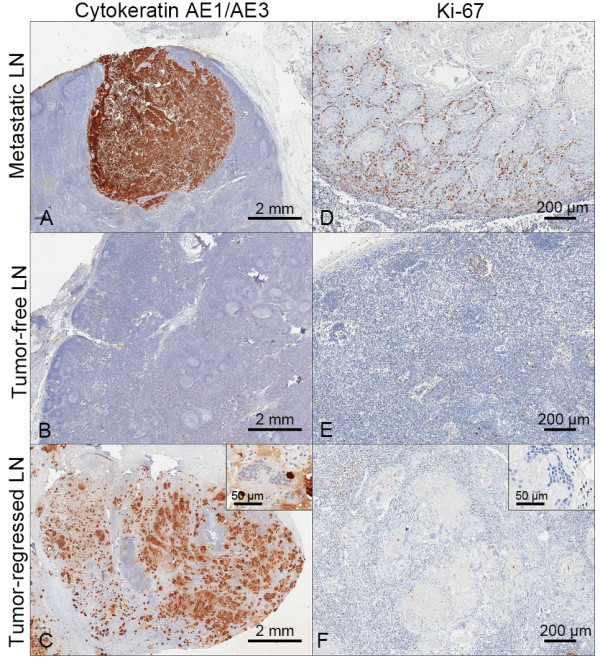
**Immunohistochemical validation of the tumor‒regressed lymph node. (A**‒**C)** Cytokeratin AE1/AE3 immunostaining. **(D-F)** Ki- 67 staining. There were no Ki-67(+) proliferating cells around the tumor-regressed remnant keratin debris (Inserts showed higher magnification views).

Complete tumor-regression in the lymph node was confirmed by cytokeratin and Ki-67 staining (Figure 2). High expression of cytokeratin and Ki-67 levels were observed in the tumor cells in metastatic nodes (Figure 2A, D), meanwhile cytokeratin expression was noted in the keratin debris and no Ki-67(+) tumor cells were found in the tumor-regressed lymph node (Figure 2C,F). In the tumor-free lymph node, the cytokeratin expression was not detected (Figure 2B). And most of the cells were CD86(+) cells with a clump of CD68(+) or CD11c(+) cells (Additional file 3: Figure S2), suggesting high infiltration of dendritic cells and macrophages in the tumor-regressed lymph nodes. However, the distribution of CD4(+), CD8(+) and CD20(+) cells were similar or relatively homogenous in the area around the metastatic and regressed nodes (data not shown).

**Figure 3:**
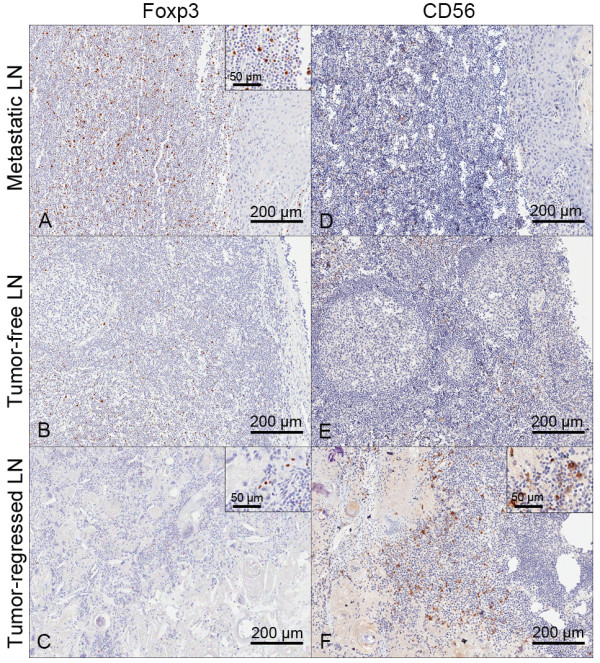
**Comparison of Foxp3 and CD56 cell distribution. (A-C)** Foxp3 staining. **(D-F)** CD56 staining. Higher density of Foxp3(+) Treg cells in the metastatic lymph nodes and more infiltration of CD56(+) NK cells in the tumor regressed lymph nodes were observed (Inserts showed higher magnification views).

Marked differences between the metastatic node and tumor-regressed node were found in the incidence of Foxp3(+) regulatory T (Treg) cells and CD56(+) natural killer (NK) cells (Figure 3). There was nearly a two-fold increase in the density of Foxp3(+) Treg cells in the metastatic node (Figure 3A), compared with the non-tumor bearing lymph node (Figure 3B). Meanwhile, very few Foxp3(+) cells were noted in the tumor-regressed lymph node (Figure 3C). These findings suggested that the immune-suppressive microenvironment of Treg cells could be reversed in the tumor-regressed lymph node. In addition, a noticeable infiltration of CD56(+) NK cells was found around the keratin debris in the tumor-regressed lymph nodes (Figure 3F); however, in the metastatic and control lymph nodes, the CD56(+) cell population was negligible (Figure 3D-E). Tumor-free lymph nodes were negative for cytokeratin, and had lower expression of Ki-67, Foxp3 and CD56 than other lymph nodes.

## Discussion

The purpose of this case report is to compare the differences of the immune cell profiles between the lymph node harboring malignant tumor cells and the tumor-regressed lymph node in the same patient, which possibly occurred through the rare process of spontaneous regression. Even though the results could not provide direct evidence of the underlying mechanisms of the spontaneous regression of the malignant tumor, our findings may have clinical significance in that the immune cell profiles according to the different disease status in the lymph nodes were analyzed in the same patient. Because the individual had quite a variable range of immune cell populations, we believe our data from the single patient may reflect the change of immunity related to tumor regression more accurately.

There was a time gap of 4 years 6 months between the initial and salvage surgery of neck lymph nodes. The pathology of the salvage surgery revealed total tumor regression and no viable tumor cells in the lymph nodes dissected. Thus, transition between tumor and non-tumor area was not observed in the specimens, suggesting that tumor cells arriving at the lymph nodes previously may undergo total cell-death process due to immunological events or unknown etiology. Of note, the tumor-regressed lymph node was located in the contralateral lower neck, which was not exposed to any treatments, such as surgery or radiation.

A previous study on the immune response in cervical intra-epithelial neoplasia with and without spontaneous regression reported that CD8(+), CD4(+)/CD25(+) cell ratios and CD138(+) plasma cells were independent predictors of tumor regression [14]. However, the study did not evaluate the spatial and temporal changes of the immune profiles in the patients. Meanwhile, our study clearly showed a feature of low Treg and high NK cells in the tumor-regressed lymph node in the same background of immunity.

There have been many reports supporting the involvement of Treg and NK cells in cancer remission. Treg cells are well known to play a major role in tumor-driven immune evasion [15-17]. Thus, there is a high chance that Treg cells can also exert their effects on tumor colonization and growth in the metastatic lymph node. Reciprocally, a decrease of Treg cells was one of the hallmarks of tumor regression in our study. NK cells are a subset of cytotoxic cells, important to the maintenance of innate immunity [18], and they seem to be a main effector cell for inducing tumor regression. Taken together, the interaction between Treg and NK cells may be an important phenomenon in spontaneous tumor regression [19].

However, the differences of immune cell profiles in our study were not able to be translated into a cause-effect relationship and the dynamics of tumor regression. For instance, a huge number of macrophages might infiltrate into tissues following malignant tumor cell death. Dendritic cells could accumulate around a regressed tumor, just after cell antigens had been exposed outside after cell death. Thus, we have to be very cautious about the interpretation of these results. Nevertheless, our study illustrated that Treg and NK cells were the main differences among various immune cell populations in the tumor-regressed lymph node, suggesting that they could modify the tumor microenvironment to suppress tumor growth and enhance self-arrest of the tumor.

## Conclusion

We describe a case of spontaneous malignant tumor regression in a metastatic lymph node, and through immunohistochemical analysis of the tumor-regressed lymph node, reveal the key features of a lower recruitment of Treg cells and a high infiltration of NK cells in this case of spontaneous regression. Thus, we think that Treg and NK cells may play a key role in tumor regression, and additionally, the modulation of Treg cells, as part of the group of NK cells, may be a good therapeutic method to control lymph node metastasis. We believe our findings should be validated through a large cohort and in-depth mechanistic study.

## Consent

Written informed consent was obtained from the patient for publication of this Case Report and any accompanying images. A copy of the written consent is available for review by the Editor-in-Chief of this journal.

## Competing interests

The authors declare that they have no competing interests.

## Authors’ contributions

NC; Clinical data collection, Literature search, Writing article, Final approval of the article. JKC; Analysis and interpretation, Final approval of the article. C-HB; Critical revision of the article, Final approval of the article. Y-HK: Pathology analysis, Final approval of the article. H-SJ; Conception and design, Literature search, Critical revision of the article, Final approval of the article. All authors read and approved the final manuscript.

## Supplementary Material

Additional file 1: Figure S1 Pre-surgical evaluation of the tumor-regressed lymph node. (A-B) CT and US strongly suggest node recurrence based on the enhancement pattern (white arrow) and irregular internal echogenicity (mark). (C-D) Aspiration cytology showed tumor recurrence due to the presence of keratin debris and anucleated squama in the aspirates (black arrows). Click here for file

Additional file 2: Table S1 Antibodies used in this study for immunostaining. Click here for file

Additional file 3: Figure S2 Staining of dendritic cells and macrophages in tumor-regressed node. (A) CD86. (B) CD68, (C) CD11c staining. Most of the cells around the keratin debris were CD86(+) cells with CD68(+) or CD11c(+) cells, suggesting high infiltration of dendritic cells and macrophage in the tumor-regressed lymph nodes. Click here for file
