# Supplementary figures and images for: Spontaneous regression of metastatic cancer cells in the lymph node: a case report
Source: BMC Res Notes. 2014 May 13;7:293. doi: 10.1186/1756-0500-7-293 (PMC4025537; doi:10.1186/1756-0500-7-293)

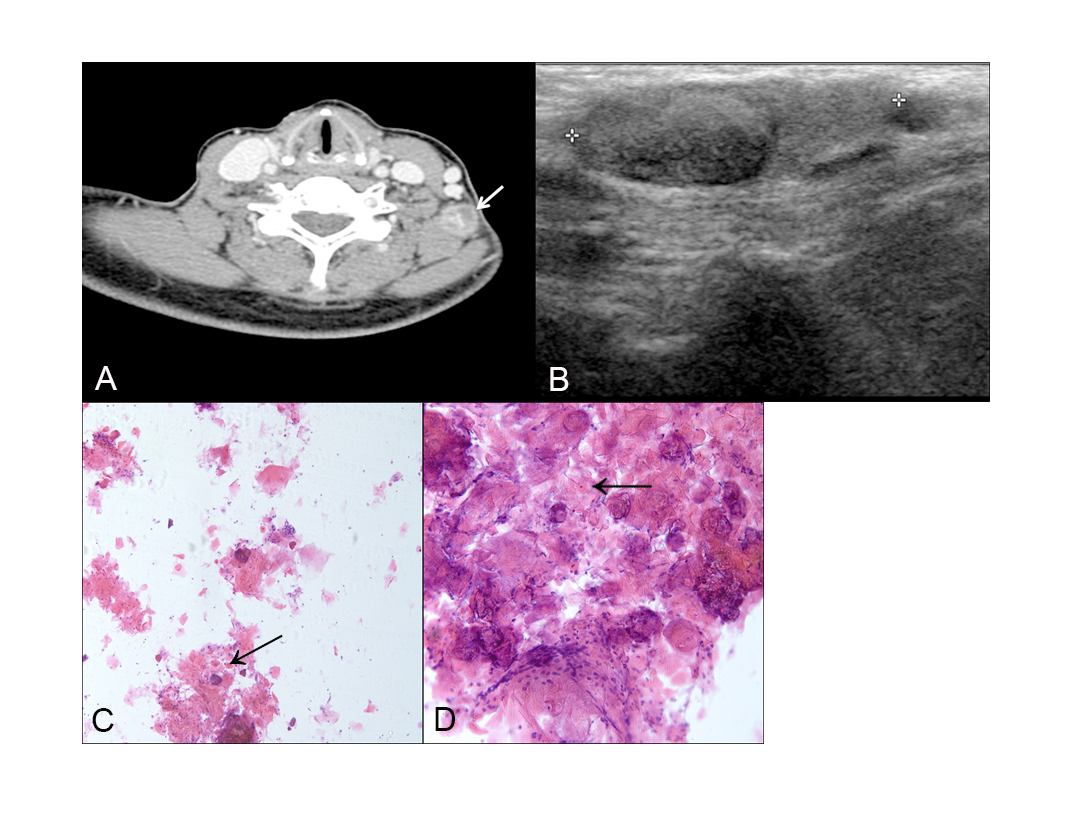

Supplement: Additional file 1: Figure S1 — Pre-surgical evaluation of the tumor-regressed lymph node. (A-B) CT and US strongly suggest node recurrence based on the enhancement pattern (white arrow) and irregular internal echogenicity (mark). (C-D) Aspiration cytology showed tumor recurrence due to the presence of keratin debris and anucleated squama in the aspirates (black arrows). [file 1756-0500-7-293-S1.tiff]

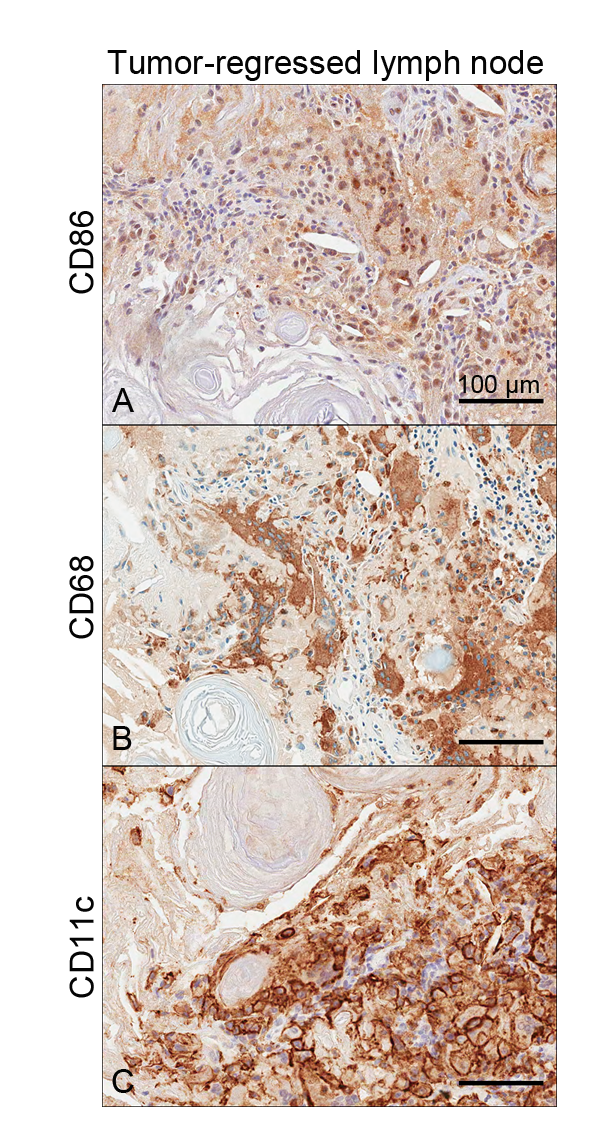

Supplement: Additional file 3: Figure S2 — Staining of dendritic cells and macrophages in tumor-regressed node. (A) CD86. (B) CD68, (C) CD11c staining. Most of the cells around the keratin debris were CD86(+) cells with CD68(+) or CD11c(+) cells, suggesting high infiltration of dendritic cells and macrophage in the tumor-regressed lymph nodes. [file 1756-0500-7-293-S3.tiff]
