# Supplementary material for: Spontaneous regression of metastatic cancer cells in the lymph node: a case report
Source: BMC Res Notes. 2014 May 13;7:293. doi: 10.1186/1756-0500-7-293 (PMC4025537; doi:10.1186/1756-0500-7-293)
Supplement: Additional file 2: Table S1 — Antibodies used in this study for immunostaining. [file 1756-0500-7-293-S2.doc]

**Supplement Table 1.** Antibodies used in this study for immunostaining

| Antibody Targets | Manufacturer | Clone | Dilution |
| --- | --- | --- | --- |
| Cytokeratin | DAKO (Carpentaria, CA) | AE1/AE3 | 1:500 |
| Ki-67 | DAKO | MIB1 | 1:200 |
|  |  |  |  |
| CD4 | NOVOCASTRA  (Leica Microsystems, Buffalo Grove, IL) | IF6 | 1:50 |
| CD8 | NOVOCASTRA | 1A5 | 1:200 |
| CD20 | NOVOCASTRA | L26 | 1:200 |
|  |  |  |  |
| CD68 | DAKO | KP-1 | 1:4000 |
| CD123 | LIFESPAN (LSBio, Seattle, WA) | 6H6 | 1:200 |
| CD11c | Abcam, (Cambridge, UK) | EP1347Y | 1:300 |
| CD86 | Abcam | EP1158Y | 1:500 |
| Foxp3 | Abcam | 236A/E7 | 1:100 |
| Mannose receptor | Abcam | 5C11 | 1:500 |
